# Prevalence and relevant factors of halitosis in Chinese subjects: a clinical research

**DOI:** 10.1186/s12903-019-0734-4

**Published:** 2019-03-13

**Authors:** Minquan Du, Leitao Li, Han Jiang, Yuqiao Zheng, Jing Zhang

**Affiliations:** 1 MOST KLOS & KLOBM, School & Hospital of Stomatology, Wuhan University, Luoyu Road 237, Wuhan City, Hubei China; 2 Xiangya Stomatology Hospital, Central South University, No. 72, Xiangya Road, Changsha, Hunan China; 3 Department of Oral Medicine, Xiangya Hospital, Central South University, No. 88, Xiangya Road, Changsha, Hunan China

**Keywords:** Halitosis, Relevant factors, Oral health status, Tongue coating

## Abstract

**Background:**

The aim of this study was to investigate the prevalence of halitosis among Chinese subjects and to analyze the associated factors that influence halitosis.

**Methods:**

This study included subjects complaining of halitosis who came to the clinic between 2014 and 2016. Questionnaires were used to obtain general information from patients. An organoleptic test was conducted, and volatile sulfur compounds measurement was obtained to assess halitosis. In conjunction with these tests, the oral health status of each patient was recorded.

**Results:**

In total, there were 205 samples entered into data analysis, and the patients’ age ranged from 18 to 71 years (mean ± SD: 32.44 ± 10.31). Of these patients, 65.9% had an organoleptic score ≥ 2, and 41% of patients had a volatile sulfur compound level ≥ 110 ppb. The findings indicated that the prevalence of halitosis was higher in males than in females (55.6% vs. 44.4%, respectively, *P* = 0.018). Several factors including the duration of bad breath, rhinitis, tongue coating and periodontal conditions were found to be associated with the organoleptic score. Tongue coating was also associated with the volatile sulfur compound level.

**Conclusions:**

Among these subjects, 65.9% had halitosis. Oral health status was strongly associated with halitosis, and tongue coating was the most important factor.

**Electronic supplementary material:**

The online version of this article (10.1186/s12903-019-0734-4) contains supplementary material, which is available to authorized users.

## Background

The term *halitosis* refers to an odor deemed unpleasant or offensive to others that emits from the oral cavity; it is also known as oral malodor or ‘bad breath’ [1]. Halitosis is divided into genuine halitosis, pseudo-halitosis and halitophobia [2]. Among these types, the latter two are caused by psychological factors of patients. Genuine halitosis includes physiologic halitosis such as morning breath, pathological halitosis (either from intra-oral or extra-oral sources) and halitosis caused by other factors such as eating garlic. While halitosis has a complex etiology, an anaerobic environment and anaerobic bacteria (mainly gram-negative bacteria) are the main causes. Volatile sulfur compound (VSC) are the major odoriferous components of halitosis, with hydrogen sulfide, methyl mercaptan, and dimethyl sulfide as the main contributors. In addition, diamines, short-chain fatty acids, and indoles contribute to halitosis [3].Halitosis is not only a reflection of the physical condition [4–6] but also has a psychological impact resulting from social isolation [7–9]. Therefore, oral malodor has been recognized as one of the most common conditions for which people seek dental care, ranking third behind tooth decay and periodontal disease [10].

Numerous studies have investigated the risk factors of halitosis. Nearly 90% of genuine halitosis originates from intra-oral sources. Tongue coating and periodontal status are some of the most important risk factors [11–15]. Other intra-oral factors such as fixed orthodontic brackets [16], dry mouth, oral mucosal diseases and oral malignancy also can result in oral malodour [3]. Nearly 5–10% of genuine halitosis originates from extra-oral sources [17], such as chronic tonsillitis [18], Helicobacter pylori infection [19], chronic renal failure [3] and metabolic disorders [20]. In addition, one study in Japan showed that social anxiety may be a causal factor of pathologic subjective halitosis [21].

There are several methods used to evaluate halitosis, such as questionnaires, organoleptic tests, and VSC monitors. Questionnaires can determine the prevalence of halitosis in the population and the influence factors related to daily habits. Questionnaires are often used in epidemiological investigations and clinical research, as they are simple to implement and require less manpower and material resources [22, 23]. Organoleptic test is the most direct way to detect halitosis and is also considered to be the gold standard of halitosis assessment. The organoleptic score (OS) presented by Rosenberg in the 1990s is still widely used around the world today [24]. The most common methods of clinically assessing VSC levels are portable gas chromatography (trade name Oral Chroma™) and the portable sulfide detector (trade name Halimeter®). Many studies have shown that both the Oral Chroma™ and Halimeter® have a certain reliability and scientific value for the diagnosis of halitosis and can be used as diagnostic tools for halitosis [25, 26]. Previous studies have shown that the organoleptic test was significantly associated with the Halimeter®; the Pearson values were 0.822 [27] and 0.74 [25]. These studies revealed that the Halimeter is a simple and reliable chair-side method of testing for halitosis.

The aim of this study was to investigate the factors related to halitosis by questionnaire survey, organoleptic test, and Halimeter® in order to assist the clinical treatment of halitosis and guide patients in effectively preventing halitosis.

## Methods

### Study participants

This study included subjects who complained of halitosis and came to the Department of Preventive Dentistry, School & Hospital of Stomatology of Wuhan University (Wuhan, China) between 2014 and 2016. Exclusion criteria included any of the following: less than 18 years of age, antibiotic therapy or periodontal treatment in the previous month, oral mucosal disease, and consumption of garlic, onions, alcohol, coffee, betel nut, tobacco or spicy food on the same day. All assessments were carried out ante meridiem. The participants were instructed to brush their teeth and eat breakfast on the examination day to distinguish oral malodor and morning breath, and we informed he participants of avoiding to use chewing gun, mouth freshener and perfume on the examination day. The patients were informed both verbally and in writing about the purpose and methods of the research, and each participant signed an informed letter of consent prior to examination.

### Questionnaire

A self-administered questionnaire was conducted in Chinese for this study (Additional file 1). To ensure the validity of the questionnaire, a pilot study was conducted on 30 people who were not included in the later study, and modifications were made accordingly to ensure the feasibility and practicality of the questionnaire.

The questionnaire was made up of 5 parts. The first part consisted of questions to collect socio demographic factors including gender, age and educational attainment. The second part was related to the participant’s personal perception of halitosis (e.g., whether the participant had been aware of their halitosis or had been informed by others; at what time it was the most serious; whether it had ever been treated; and the relationship of halitosis to oral diseases, systemic diseases and physical health), and the duration and social impacts of halitosis. Lifestyle factors were included in the third part (e.g., smoking and drinking habits, preference for sweets and spicy foods, xerostomia experience and psychological stress experience). The fourth part was concerned with some oral hygiene behaviors (e.g., date of last dental visit, frequency of brushing teeth, utilization of special mouthwash, and tongue cleaning). The last part asked about systemic diseases (e.g., gastroesophageal reflux, gastroenteritis, belching, constipation, rhinitis, tracheitis, nephropathy, hepatopathy, and diabetes).

### Clinical examination

Clinical examinations and halitosis assessments were performed by the same trained examiner under the guidance of Professor Du. First, halitosis was measured by organoleptic test using the plastic tube method. A plastic tube was inserted into patient’s mouth, and they were instructed to exhale slowly while the dentist assessed the odor at the other end of the tube. The organoleptic score (OS), presented by Rosenberg in 1992, was utilized to assess the intensity. Six degrees were included in this criterion: 0 = absence of odor, 1 = barely noticeable odor, 2 = slight malodor, 3 = moderate malodor, 4 = strong malodor and 5 = severe malodor [24]. In this study, an OS greater than or equal to 2 was diagnosed as halitosis [28]. To test the intra-examiner reliability during the survey period, 10% of the patients were randomly chosen to be remeasured. The intra-examiner kappa value for OS was 0.71.

Next, halitosis was also assessed by a VSC monitor termed ‘Halimeter®’ (Interscan, Chatsworth CA, USA). Every patient was asked to keep his or her mouth closed for at least 1 min before the assessment. A disposable plastic tube connected the sampling pedestal to the patient’s mouth. During the assessment, the patient was instructed to hold the tube above the posterior part of the dorsum of the tongue without touching the lingual papilla or mucosa. It was necessary for the patient to keep their mouth slightly opened and breathe through their nose. The final value displayed in parts per billion (ppb) was recorded. A VSC level greater than or equal to 110 ppb was diagnosed as halitosis according to the manufacturer instructions.

Finally, the oral health status of each patient was assessed by noting the presence of dental caries, residual crowns/roots, periodontal status, tongue coating, and restorations. The standard of dental caries was set according to the criteria suggested by the World Health Organization (WHO) Basic Methods for Oral Health Surveys 4th edition [29]. Only tooth decay was recorded, while missing and filled teeth were not included. The term residual crown indicates that more than half of the crown was destroyed because of tooth decay or other causes. If all or nearly all of the crown is lost, it is called a residual root. Periodontal status was measured by the community periodontal index (CPI). Ten index teeth (17, 16, 11, 26, 27, 36, 37, 31, 46, 47) were examined in patients more than 20 years old, and six index teeth (16, 11, 26, 36, 31, 46) were examined in patients between 18 to 20 years old. Each tooth was examined using the six-point method and diagnosed according to CPI criteria from 0 to 4: 0 = health periodontal conditions; 1 = gingival bleeding on probing; 2 = calculus and bleeding; 3 = periodontal pocket 4–5 mm; 4 = periodontal pocket ≥6 mm [30].

In this study, the area and thickness of tongue coating were scored separately. This study utilized a modified version of the tongue coating score presented by Rosenberg [31]: (1) area of tongue coating: 0 = no coating, 1 = coating less than one third of tongue, 2 = coating between one-third and two-thirds of the tongue, 3 = coating more than two-thirds of the tongue; (2) thickness of tongue coating: 0 = no coating, 1 = thin coating, the lingual papilla was clearly visible, 2 = moderate coating, the lingual papilla was faintly visible, 3 = abundant coating, the lingual papilla was covered and invisible. The prostheses could be divided into fixed denture and movable denture, and were accordingly recorded as such. The intra-examiner kappa value on dental caries, CPI score, and tongue coating was 0.93, 0.83, and 0.82, respectively.

### Statistical analysis

SPSS, version 21.0 (IBM, Armonk, NY, USA) was used to perform the statistical analyses. The OS and VSC value were dichotomized as 0 (OS < 2, or VSCs < 110 ppb) and 1 (OS ≥2, or VSCs ≥110 ppb). The prevalence of halitosis was the proportion of participants with OS ≥2. For further descriptive statistical analysis of the independent variables, percentages were calculated. The relationship between oral malodor and the questionnaire items and oral health status was evaluated by chi-square test and binary logistic regression. The dependent variables were defined as OS ≥2 or VSCs ≥110 ppb. All independent variables found to be significant by chi-square test were entered as candidates and included in the binary logistic regression analysis. Odds ratios (OR) with 95% confidence intervals (CI) were calculated in the logistic regression model to evaluate the connection between the dependent variables and the potential influence factors. In addition, the Pearson correlation coefficient was used to assess the correlation between organoleptic test and Halimeter®. All levels of statistical significance were set at 0.05.

## Results

From April 2014 to January 2016, a total of 219 subjectscomplaining of halitosis came to the Department of Preventive Dentistry, School & Hospital of Stomatology, Wuhan University (Wuhan, China.). Of these patients, 9 patients with incomplete data and 5 patients under 18 years old were excluded, and 205 patients entered the result analysis stage. Among those patients, more females than males visited the clinic (50.7% vs. 49.3%, respectively). The results showed that 65.9% of the subjects had OS ≥2, while 34.1% of the patients had no or slight odor (pseudo-halitosis or halitophobia), and 41.0% had a VSC level ≥ 110 ppb. The percentage of OS ≥2 in males was significantly higher than in females (55.6% vs. 44.4%, respectively, *P* = 0.018). However, gender showed no relationship with the VSC level. The patients’ age ranged from 18 to 71 years (mean ± SD: 32.44 ± 10.31). The highest proportion of patients was in the 18–29 years old group (47.8%), followed by the 30–40 years old group (33.7%). With regard to education, 10.2% of the participants had an education level of junior high school, 40.0% had graduated from high school or technical college, 41.5% were university undergraduates or had a bachelor’s degree, and 8.3% had a master’s degree or above. In addition, there were no significant differences in the prevalence of halitosis according to age and educational attainment (Table 1). The correlation analysis results of the two methods indicated that the organoleptic test was significantly correlated with Halimeter® (r = 0.349, *P* < 0.001).

**Table 1:** The general characteristics of the study group

	N (%)	OS≥2 (%)	P	VSC ≥ 110 (%)	P
Gender			0.018		0.394
Male	101 (49.3)	75 (55.6)		38 (45.2)	
Female	104 (50.7)	60 (44.4)		46 (54.8)	
Total	205	135 (65.9)		84 (41.0)	
Age			0.105		0.448
18–29	98 (47.8)	60 (61.2)		42 (42.9)	
30–40	69 (33.7)	44 (63.8)		23 (33.3)	
41–50	24 (11.7)	18 (75.0)		11 (45.8)	
51–60	11 (5.4)	11 (100.0)		6 (54.5)	
> 60	3 (1.5)	2 (66.7)		2 (66.7)	
Total	205	135 (65.9)		84 (41.0)	
Educational attainment			0.774		0.196
Junior high school	21 (10.2)	16 (76.2)		13 (61.9)	
High school or technical college	82 (40.0)	53 (64.6)		30 (36.6)	
College student	85 (41.5)	55 (64.7)		35 (41.2)	
Master’s degree or above	17 (8.3)	11 (64.7)		6 (35.3)	
Total	205	135 (65.9)		84 (41.0)	

Tables 2 shows the associations between oral malodor assessments (OS and VSC level) and personal perception assessed by chi-square test. The overwhelming majority (94.1%) of those patients was self-reported halitosis, and 88.1% had ever been told by others that they had bad breath. With regard to the most serious time of halitosis, 36.6% of the participants felt that it was severe all day long, 26.3% reported it was most severe in the morning, and 19.0% felt it was severe after a period of silence. Nearly one in three had halitosis for more than 3 years, and the duration of oral malodor was significantly associated with the occurrence of OS ≥2 (*P* = 0.006). Among the study participants, 70.7% thought bad breath had great negative impacts on social communication, whereas only 37.1% of the subjects consulted doctors and received treatment regarding their conditions. Furthermore, most of the patients agreed that halitosis is related to oral diseases (74.6%), systemic diseases (68.4%) and physical health (86.8%).

**Table 2:** Chi-square test for relationship of OS and VSC level and personal perception

	N (%)	OS≥2 (%)	P	VSC ≥ 110 (%)	P
Self-reported halitosis			0.548		0.237
Yes	193(94.1)	128(66.3)		77(39.9)	
No	12(5.9)	7(58.3)		7(58.3)	
Total	205	135(65.9)		84(41.0)	
Informed by others			0.070		1.000
Yes	180(88.1)	123(68.3)		74(41.1)	
No	25(11.9)	12(48.0)		10(40.0)	
Total	205	135(65.9)		84(41.0)	
Most serious time			0.121		0.217
In the morning	54(26.3)	37(68.5)		23(42.6)	
Whole day	75(36.6)	55(73.3)		34(45.3)	
Pressured	15(7.3)	8(53.3)		2(13.3)	
Menstrual period	5(2.4)	2(40.0)		2(40.0)	
Hunger	9(4.4)	8(88.9)		6(66.7)	
After silence	39(19.0)	21(53.8)		14(35.9)	
Other time	8(3.9)	4(50.0)		3(37.5)	
Total	205	135(65.9)		84(41.0)	
Have you ever been treated for halitosis			0.545		0.304
Yes	76(37.1)	48(63.2)		35(46.1)	
No	129(62.9)	87(67.4)		49(38)	
Total	205	135(65.9)		84(41.0)	
Halitosis related to oral diseases			0.793		0.916
Yes	153(74.6)	101(66.0)		63(41.2)	
No	4(2.0)	2(50.0)		2(50.0)	
Do not know	48(23.4)	32(66.7)		19(39.6)	
total	205	135(65.9)		84(41.0)	
Halitosis related to systemic diseases			0.272		0.362
Yes	140(68.3)	88(62.9)		53(37.9)	
No	13(6.3)	8(61.5)		7(53.8)	
Do not know	52(25.4)	39(75.0)		24(46.2)	
total	205	135(65.9)		84(41.0)	
Halitosis related to physical health			0.157		0.566
Yes	178(86.8)	113(63.5)		71(39.9)	
No	3(1.5)	2(66.7)		2(66.7)	
Do not know	24(11.7)	20(83.3)		11(45.8)	
Total	205	135(65.9)		84(41.0)	
Duration (years)			0.006		0.457
< 1	43(21.0)	20(46.5)		16(37.2)	
1–3	48(23.4)	29(60.4)		18(37.5)	
> 3	67(32.7)	51(76.1)		26(38.8)	
Do not know	47(22.9)	35(74.5)		24(51.1)	
Total	205	135(65.9)		84(41.0)	
Social impacts of halitosis			0.130		0.357
Great seriously affected	145(70.7)	98(67.6)		55(37.9)	
Moderate affected	34(16.6)	17(50.0)		17(50.0)	
A little affected	25(12.2)	19(76.0)		11(44.0)	
No affected	1(0.5)	1(100.0)		1(100.0)	
Total	205	135(65.9)		84(41.0)	

Table 3 presents the relationship between oral malodor and lifestyle factors. Among the study group, 13.7% were smokers, 23.9% were regular wine drinkers, and 47.3% had a preference for sweets, while 43.3% often felt thirsty, and 18.0% often had psychological stress. It is worth noting that the VSC level was significantly associated with sweets consumption (*P* = 0.033), xerostomia(*P* = 0.022) and psychological stress (*P* = 0.023) by chi-square test.

**Table 3:** Chi-square test for relationship of OS and VSC level and life style factors

	N (%)	OS≥2 (%)	P	VSC ≥ 110 (%)	P
Smoking			0.463		0.356
Yes	28(13.7)	21(75.0)		8(28.6)	
No	163(79.5)	104(63.8)		70(42.9)	
Used to smoke	14(6.8)	10(71.4)		6(42.9)	
Total	205	135(65.9)		84(41.0)	
Drinking			0.213		0.111
Yes	49(23.9)	36(73.5)		16(32.7)	
No	140(68.3)	91(65.0)		64(45.7)	
Used to drink	16(7.8)	8(50.0)		4(25.0)	
Total	205	135(65.9)		84(41.0)	
Preference to sweetmeat			0.423		0.033
Yes	97(47.3)	60(61.9)		43(44.3)	
No	95(46.3)	67(79.5)		32(33.7)	
Used to	13(6.3)	8(61.5)		9(69.2)	
Total	205	135(65.9)		84(41.0)	
Preference to spicy foods (garlic, onion, etc.)			0.588		0.564
Often	13(6.3)	8(61.5)		7(53.8)	
Sometimes	91(44.4)	57(62.6)		35(38.5)	
Only as seasoning	101(49.3)	70(69.3)		42(41.6)	
Total	205	135(65.9)		84(41.0)	
Xerostomia experience			0.301		0.022
Often	89(43.4)	55(61.8)		28(31.5)	
Sometimes	116(56.6)	80(69.0)		56(48.3)	
Total	205	135(65.9)		84(41.0)	
Psychological stress experience			0.134		0.023
Often	37(18.0)	25(67.6)		11(29.7)	
Sometimes	134(65.4)	82(61.2)		52(38.8)	
Recently	9(4.4)	7(77.8)		7(77.8)	
Never	25(12.2)	21(84.0)		14(56.0)	
Total	205	135(65.9)		84(41.0)	

Regarding the oral hygiene behaviors of the participants, 51 people had visited a dentist within the last year, while 77 people had never visited a dentist. Most people (71.7%) brushed their teeth twice a day, 8.3% brushed three times and 20% brushed once or less. Among the study sample, only 15.6% used a mouth rinse, and 26.3% tried to clean their tongues. With respect to the oral hygiene practices, the last dental visit (*P* = 0.025) and tooth brushing frequency (*P* = 0.026) were significantly associated with the OS (Table 4).

**Table 4:** Chi-square test for relationship of OS and VSC level and oral hygiene behaviors

	N (%)	OS≥2 (%)	P	VSC ≥ 110 (%)	P
Time of last dental visit			0.025		0.417
less than 1 year	51(24.9)	26(51.0)		17(33.3)	
1 year ago	38(18.5)	22(57.9)		15(39.5)	
2 years ago	11(5.4)	7(63.6)		4(36.4)	
3 years ago	28(13.7)	21(75.0)		10(35.7)	
Never	77(37.6)	59(76.6)		38(49.4)	
Total	205	135(65.9)		84(41.0)	
Toothbrushing frequency			0.026		0.365
3 times	17(8.3)	9(52.9)		5(29.4)	
Twice	147(71.7)	92(62.6)		59(40.1)	
Once or less	41(20.0)	34(82.9)		20(48.8)	
Total	205	135(65.9)		84(41.0)	
Utilization of mouthwash			0.591		0.676
Yes	32(15.6)	20(62.5)		12(37.5)	
No	163(79.5)	107(65.6)		69(42.3)	
Used to	10(4.9)	8(80.0)		3(30.0)	
Total	205	135(65.9)		84(41.0)	
Tongue cleaning			0.246		0.109
Yes	54(26.3)	32(59.3)		17(31.5)	
No	151(73.7)	103(68.2)		67(44.4)	
Total	205	135(65.9)		84(41.0)	

Table 5 presents the relationship between halitosis and systemic diseases, only rhinitis showed a statistically significant relationship with OS (*P* = 0.049). Table 6 shows the association between the percentage of patients with halitosis and their oral health status. The OS was associated with thickness of tongue coating (*P* < 0.001), area of tongue coating (*P* = 0.002), and CPI score (*P* < 0.001). The VSC level was associated with residual crowns/roots (*P* = 0.010), thickness of tongue coating (P = 0.010) and area of tongue coating (*P* = 0.044).

**Table 5:** Chi-square test for relationship of OS and VSC level and systemic diseases

	N (%)	OS≥2 (%)	P	VSC ≥ 110 (%)	P
Gastroesophageal reflux			0.767		1.000
Yes	13(6.3)	8(61.5)		5(38.5)	
No	192(93.7)	127(66.1)		79(41.1)	
Total	205	135(65.9)		84(41.0)	
Gastroenteritis			0.533		0.424
Yes	30(14.6)	18(60.0)		10(33.3)	
No	175(85.4)	117(66.9)		74(42.3)	
Total	205	135(65.9)		84(41.0)	
Belching			0.721		0.740
Yes	9(4.4)	7(77.8)		3(33.3)	
No	196(95.6)	128(65.3)		81(41.3)	
Total	205	135(65.9)		84(41.0)	
Constipation			0.376		1.000
Yes	45(22.0)	27(60.0)		18(40.0)	
No	160(78.0)	108(67.5)		66(41.3)	
Total	205	135(65.9)		84(41.0)	
Rhinitis			0.049		0.562
Yes	78(38.0)	58(74.4)		34(43.6)	
No	127(62.0)	77(60.6)		50(39.4)	
Total	205	135(65.9)		84(41.0)	
Trachitis			0.346		0.366
Yes	12(5.9)	6(50.0)		3(25.0)	
No	193(94.1)	129(66.8)		81(42.0)	
Total	205	135(65.9)		84(41.0)	
Nephropathy			1.000		0.307
Yes	4(2.0)	3(75.0)		3(75.0)	
No	201(98.0)	132(65.7)		81(40.3)	
Total	205	135(65.9)		84(41.0)	
Hepatopathy			0.272		0.798
Yes	16(7.8)	13(81.3)		7(43.8)	
No	189(92.2)	122(64.6)		77(40.7)	
Total	205	135(65.9)		84(41.0)	
Diabetes			0.301		1.000
Yes	4(2.0)	4(100.0)		2(50.0)	
No	201(98.0)	131(65.2)		82(40.8)	
Total	205	135(65.9)		84(41.0)

**Table 6:** Chi-square test for relationship of OS and VSC level and oral health status

	N (%)	OS≥2 (%)	P	VSC ≥ 110 (%)	P
Dental caries			0.364		1.000
Yes	42(20.5)	25(59.5)		17(40.5)	
No	163(79.5)	110(67.5)		67(41.1)	
Total	205	135(65.9)		84(41.0)	
Residual crowns/roots			0.101		0.045
Yes	23(11.2)	19(82.6)		14(60.9)	
No	182(88.8)	116(63.7)		70(38.5)	
Total	205	135(65.9)		84(41.0)	
CPI score			< 0.001		0.426
0	9(4.4)	3(33.3)		2(22.2)	
1	48(23.4)	21(43.8)		16(33.3)	
2	129(62.9)	95(73.6)		56(43.4)	
3	17(8.3)	15(88.2)		9(52.9)	
4	2(1.0)	1(50.0)		1(50.0)	
Total	205	135(65.9)		84(41.0)	
Thickness of tongue coating			< 0.001		0.010
0	18(8.8)	8(44.4)		6(33.3)	
1	78(38.0)	41(52.6)		22(28.2)	
2	53(25.9)	41(77.4)		25(47.2)	
3	56(27.3)	45(80.4)		31(55.4)	
Total	205	135(65.9)		84(41.0)	
Area of tongue coating			0.002		0.044
0	17(8.3)	7(41.2)		5(29.4)	
1	58(28.3)	30(51.7)		21(36.2)	
2	69(33.7)	51(73.9)		24(34.8)	
3	61(29.8)	47(77.0)		34(55.7)	
Total	205	135(65.9)		84(41.0)	
Restorations			0.051		0.373
No	183(89.3)	125(68.3)		76(41.5)	
Fixed denture	21(10.2)	9(42.9)		7(33.3)	
Movable denture	1(0.5)	1(100.0)		1(100.0)	
Total	205	135(65.9)		84(41.0)	

Table 7 shows the results of binary logistic regression analysis for the OS (OS ≥2), and Table 8 shows the VSC values (VSCs ≥110 ppb). Only statistically significant associations are presented. The occurrence of an OS ≥2 was significantly associated with longer duration of halitosis (OR = 4.728, *p* = 0.014), the existence of rhinitis (OR = 2.100, *P* = 0.045), thicker tongue coating (OR = 4.358, *P* = 0.001), and higher CPI score (OR = 11.911, P < 0.001). In addition, subjects with a thicker tongue coating also tended to have a high likelihood of a VSC level ≥ 110 ppb (OR = 2.299, *P* = 0.040).

**Table 7:** Logistic regression analysis of odds for OS ≥2

	P	OR	95 % CI	
			Lower Upper	
Duration (years)				
< 1	0.014	1	1	
1–3	0.305	1.672	0.626	4.461
> 3	0.002	4.728	1.780	12.559
Do not know	0.083	2.480	0.888	6.928
Rhinitis	0.045			
No		1	1	
Yes		2.100	1.017	4.335
Thickness of tongue coating				
0	0.001	1	1	
1	0.763	0.822	0.232	2.922
2	0.107	3.034	0.788	11.682
3	0.034	4.358	1.116	17.021
CPI score				
0	< 0.001	1	1	
1	0.449	2.065	0.316	13.496
2	0.020	9.122	1.418	55.676
3	0.005	29.611	2.746	319.331
4	0.162	11.911	0.371	382.273

**Table 8:** Logistic regression analysis of odds for VSC level ≥ 110 ppb

	P	OR	95%CI	
			Lower Upper	
Thickness of tongue coating				
0	0.040	1	1	
1	0.694	0.800	0.263	2.433
2	0.465	1.537	0.485	4.870
3	0.148	2.299	0.743	7.112

## Discussion

There have been numerous studies conducted worldwide on the prevalence of halitosis. However, most of the study samples were the general population. In Korea, an internet-based questionnaire survey indicated that the prevalence of halitosis in adults was 23.6% [32]. In Japan and China, the prevalence was 20% [33] and 27.5% [34] respectively in general population. These are much lower rates than our study reported (65.9%), which may be due to differences in the study samples. However, in similar study based on outpatient samples, this figure is comparable to the result reported in Thailand (61.1%) [13], but it is lower than that of patients in Shanghai, China (77.3%) [11]. In this study, the prevalence of halitosis (OS ≥2) was 65.9%, while the remaining patients had no or slight odor (pseudo-halitosis or halitophobia). This maybe because of that the majority of patients’ own assessment was higher than the organoleptic assessment [35].

In this study, 21.0% of subjects had complaints of halitosis for less than 1 year, 23.4% for between 1 to 3 years, and 32.7% for over 3 years. Moreover, the odds ratios for the duration of halitosis between 1 to 3 years and more than 3 years compared with that for a duration of less than 1 year were 1.672 and 4.728, respectively. It is possible that this result is because a longer duration of halitosis results in chronic and pathologic bad breath and is always accompanied by chronic conditions such as tooth decay and periodontal disease. As no previous literature on this topic has been reported, more research is needed in the future.

The present study also revealed that rhinitis was related to a higher OS. This result is corroborated by other studies that observed a relationship between ear, nose and throat (ENT) diseases and halitosis [36, 37]. Furthermore, a review published by Curd ML Bollen et al. points out that a maximum of 10% of oral malodor originates from the ENT region [20]. The main causes of halitosis among rhinitis patients are related to decomposition of organic material and putrefaction of amino acids by anaerobic proteolytic bacteria; these processes increase the production of VSC in paranasal sinuses and lead to the presence of odoriferous gases in the air expelled from the oral cavity and the nose. Additionally, foreign bodies in the nose can become a hub for bacterial degradation and hence produce an unpleasant odor to the breath [10].

An accumulation of evidence regarding halitosis and tongue coating has also been reported. There are numerous anaerobic bacteria, deciduous epithelial cells, leukocytes, and food debris in the tongue coating. The anaerobic bacteria produce a large amount of VSC by degradation of these substrates, which results in halitosis. One investigation in Belgium showed that in 2000 patients with halitosis, 76% had oral causes, with the most prevalent factor being tongue coating (43%) [17]. In the literature, the influence of tongue coating on halitosis has been determined, with a higher risk of OS ≥2 and VSC level ≥ 110 ppb found to be associated with a thicker tongue coating. However, no significant relationship was found between the area of tongue coating and OS and VSC level in the present study. This finding is in agreement with the results of a previous investigation by Chen [12]. Some researchers have indicated that halitosis is related to the amount of bacteria present on the tongue and have shown that the load of microbes in the mouth (such as the thickness or aerial density of biofilms) is the most important feature of chronic halitosis [38]. Furthermore, a previous study found the coating at the posterior dorsum of the tongue to be primarily responsible for the presence of halitosis [39], and it is difficult to remove because of the gagging reflex [40]. Therefore, it is commonly found that a tongue coating covers less than 1/3 of the tongue but completely covers the lingual papilla in halitosis patients. These studies provide critical support for our results.

There is substantial evidence indicating that poor periodontal condition is one of the main causes of halitosis [15, 41, 42]. Researchers have found that the OS and VSC level were significantly associated with bleeding index (BI) and plaque index (PLI) [43]; others have revealed that the amount of tongue coating and bleeding on probing played the most important role in increasing the concentration of VSC, followed by periodontal status, plaque index, and calculus component [44]. In turn, there is evidence showing that periodontal treatment results in a remarkable decrease in odoriferous gases [45]. In this study, CPI was used to evaluate periodontal status. The results indicated that the patients with a higher CPI score had a high likelihood of having an OS≥2, which is consistent with the results of previous investigations. Nevertheless, the CPI showed no relationship with the VSC level. It is not clear whether this result was because the previous studies utilized various indices and evaluated periodontal status more accurately. However, the CPI used in this study was originally developed by the WHO to measure community oral health, which is a broad indicator. Thus, the relationship between halitosis and periodontal status demands more detailed research in the future.

The study had limitations because the participants were patients with self-perceived halitosis, and as we discussed previously, the prevalence of halitosis was higher than that in the normal population. However, this sample did not influence the examination of risk factors because our negative control group did not have halitosis. Similar articles have been published previously [11, 13]. Moreover, no relationship was found between xerostomia and halitosis in our study. This result is consistent with the findings reported in Thai [13]. However, some studies have indicated that dry mouth can lead to an increase in VSC [46]. The reason for this difference may be that we only conducted a questionnaire survey on dry mouth rather than a clinical assessment. Some scholars noted that asking a patient’s subjective feelings and visually inspecting oral tissues are insufficient to determine whether the patient has hyposalivation. They recommended measuring the unstimulated salivary flow rate [47]. The normal unstimulated salivary flow rate is approximately 0.3–0.4 mL/min [48]. A diagnosis of hyposalivation is made when the unstimulated salivary flow rate is ≤0.1 mL/min [49]. We will improve our research and quantify dry mouth in the future. The proportion of people with gastroesophageal reflux, belching, tracheitis, nephropathy, hepatopathy, and diabetes does not exceed 10% in Table 5. These systematic diseases did not exhibit a relationship with halitosis due to the small sample size. The sample size needs to be increased in future studies.

## Conclusions

In conclusion, this study showed halitosis was a significant problem in the population studied. Additionally, the study confirmed that the etiology of halitosis is multifactorial. Longer duration, rhinitis, a thicker tongue coating, and poor periodontal conditions increased the risk of halitosis. Moreover, a thicker tongue coating increased the concentration of VSC. Professional dentists should pay attention to these aspects in clinical treatment.

## Additional file

Additional file 1 The self-administered questionnaire for this study. (DOCX 17 kb)

## Abbreviations

BI: Bleeding index
CI: Confidence intervals
CPI: Community periodontal index
ENT: Ear, nose and throat
OR: Odds ratios
OS: Organoleptic score
PLI: Plaque index
VSC: Volatile sulfur compound
WHO: World Health Organization
