# Supplementary material for: Prevalence and relevant factors of halitosis in Chinese subjects: a clinical research
Source: BMC Oral Health. 2019 Mar 13;19:45. doi: 10.1186/s12903-019-0734-4 (PMC6417129; doi:10.1186/s12903-019-0734-4)
Supplement: Supplementary file 1 — The self-administered questionnaire for this study. (DOCX 17 kb) [file 12903_2019_734_MOESM1_ESM.docx]

NO.

**THE QUESTIONNAAIRE OF HALITOSIS**

Name: Gender: Age: Phone number:

1. Your educational attainment

A. Junior high school B. High school or technical college C. College student D. Master’s degree or above

2. Have you ever been aware of your halitosis?

A. Yes B. No

3. Has anyone else told you that you have halitosis?

A. Yes B. No

4. When did you feel the halitosis most serious?

A. Weak up in the morning B. Whole day C. Pressured D. Menstrual period

E. Hunger F. After silence G. Other time

5. Have you ever been treated for halitosis?

A. Yes B. No

6. Do you think halitosis is related to oral diseases?

A. Yes B. No C. Not clear

7. Do you think halitosis is related to systemic diseases?

A. Yes B. No C. Not clear

8. Do you think halitosis is related to physical health?

A. Yes B. No C. Not clear

9. How long have you realized that or someone else tells you that you have had halitosis?

A. < 1 year B. 1-3 years C. > 3 years D. Do not know

10. Do you think halitosis will affect social interaction?

A. Great seriously affected B. Moderate affected C. A little affected D. No affected

11. Do you smoke?

A. Yes B. No C. Used to smoke

12. Do you drink?

A. Yes B. No C. Used to drink

13. Do you like sweets?

A. Yes B. No C. Used to

14. How often do you eat spicy food (garlic, onion, etc.)?

A. Often B. Sometimes C. Only as seasoning

15. How often do you have a xerostomia experience?

A. Often B. Sometimes

16. How often do you feel psychological pressure?

A. Often B. Sometimes C. Recently D. Never

17. When was the last time you visited the dentist?

A. Less than 1 year B. 1 year ago C. 2 years ago D. 3 years ago E. Never

18. How often do you brush your teeth every day?

A. 3 times B. Twice C. Once or less

19. Do you use mouthwash?

A. Yes B. No C. Used to

20. Do you clean your tongue?

A. Yes B. No

21. Do you have any of the following system diseases?

A. Gastroesophageal reflux B. Gastroenteritis C. Belching D. Constripation

E. Rhinitis F. Trachitis G. Nephropathy H. Hepatopathy I. Diabetes

You have completed this survey, thank you for your cooperation.
